# Immunoinformatics design of B and T-cell epitope-based SARS-CoV-2 peptide vaccination

**DOI:** 10.3389/fimmu.2022.1001430

**Published:** 2023-01-04

**Authors:** Muhammad Shehzad Khan, Ibrar Muhammad Khan, Syed Umair Ahmad, Ishrat Rahman, Muhammad Zahoor Khan, Muhammad Shah Zeb Khan, Zain Abbas, Shumaila Noreen, Yong Liu

**Affiliations:** ^1^ Anhui Province Key Laboratory of Embryo Development and Reproduction Regulation, Anhui Province Key Laboratory of Environmental Hormone and Reproduction, School of Biological and Food Engineering, Fuyang Normal University, Fuyang, China; ^2^ Department of Physics, College of Science, City University of Hong Kong, Kowloon, Hong Kong SAR, China; ^3^ Department of Bioinformatics Hazara University Mansehra, Mansehra, Pakistan; ^4^ Department of Basic Dental Sciences, College of Dentistry, Princess Nourah Bint Abdulrahman University, Riyadh, Saudi Arabia; ^5^ Department of Animal Breeding and Genetics, Faculty of Veterinary and Animal Sciences, University of Agriculture, Dera Ismail Khan, Pakistan; ^6^ State Key Laboratory of Animal Nutrition, Beijing Engineering Technology Research Center of Raw Milk Quality and Safety Control, College of Animal Science and Technology, China Agricultural University, Beijing, China; ^7^ Department of Biotechnology, University of Science and Technology of Bannu, Bannu, Pakistan; ^8^ School of Biomedical Science and Biomedical Engineering, Southeast University, Nanjing, China; ^9^ Department of Life Sciences, University of Management and Technology, Lahore, Pakistan; ^10^ Department of Zoology, Hazara University, Mansehra, Pakistan

**Keywords:** SARS-CoV-2, COVID-19, vaccine designing, vacciome, docking

## Abstract

SARS-COV-2 is a virulent respiratory virus, first identified in China (Wuhan) at the end of 2019. Scientists and researchers are trying to find any possible solution to this deadly viral disease. Different drug source agents have been identified, including western medicine, natural products, and traditional Chinese medicine. They have the potential to counteract COVID-19. This virus immediately affects the liver and causes a decrease in oxygen levels. In this study, multiple vacciome approaches were employed for designing a multi-epitope subunit vaccine for battling against SARS-COV-2. Vaccine designing, immunogenicity, allergenic, and physico-chemical assessment were performed by using the vacciome approach. The vaccine design is likely to be antigenic and produce potent interactions with ACE2 and NSP3 receptors. The developed vaccine has also been given to in-silico cloning models and immune response predictions. A total number of 12 CTL and 12 HTL antigenic epitopes were predicted from three selected covid-19 virulent proteins (spike protein, nucleocapsid protein, and membrane proteins, respectively) based on C-terminal cleavage and MHC binding scores. These predicted epitopes were amalgamated by AYY and GPGPG linkers, and a β-defensins adjuvant was inserted into the N-terminus of this vaccine. This analysis shows that the recommended vaccine can produce immune responses against SARS-COV-2. Designing and developing of the mentioned vaccine will require further experimental validation.

## Introduction

The SARS-CoV-2 virus (formerly 2019-nCoV) broke out in Wuhan, China, in December 2019 and circulated rapidly around the world, mainly in China, Italy, Germany, Japan, the USA, Iran, Pakistan, UAE, India, South Korea, and Russia. According to WHO, On 19 September 2022, 612,236,677 confirmed cases of coronavirus 2019 (COVID-19), and 6,514,397 deaths were reported. Scientists are struggling to find a solution to treat this deadly disease. To date more than 30 agents has been identified, including natural products, western medicine, and traditional Chinese medicine, which may be effective against COVID-19 (1).

Coronaviruses are positive-stranded RNA viruses that consist of a large genome among all viral RNAs, ranging from 27 to 32 kb. Similarly, it places inside a helical capsid made of nucleocapsid (N) protein and surrounded by an envelope. The three structural proteins are referred to as membrane protein (M), envelope protein (E), and spike protein (S). Proteins M and E are involved in the viral organization, while S-proteins invade viral cells in host cells. Some coronaviruses also contain proteins associated with the envelope of haemagglutinin esterase protein (HE). Of all the synthetic proteins, the spike forms the great speculation from the viral segment that made coronaviruses look like crowns (hence its name; corona in Latin means crown) (2).

Concerning health threats from coronaviruses, are permanent and long-lasting. Understanding the behavior of coronaviruses and controlling their spread, have vital effects on global health and economic stability (3). Coronaviruses are a member of the family Coronaviridae in the order of Nidovirales (4). However, they can divide into four categories: Alpha - coronavirus, Beta-coronavirus, Gamma-coronavirus, and Delta-coronavirus. Alpha-and beta-coronaviruses infect mammals, gamma-coronaviruses infect bird species, and delta infects both mammals and birds. Coronaviruses are classified in the order Nidovirales within the family Coronaviridae and the subfamily Coronavirinae. In the beginning, the classification of members of this subfamily was determined by their serological connections, but now it is determined by meeting a certain amount of sequence identity in a few replicase areas. This new taxonomic revision is a stark contrast to the older method (the pp1ab polyprotein and the ORF1ab gene). In accordance with these criteria, the family Coronavirinae is subdivided into four genera, namely alpha-, beta-, gamma-, and deltacoronavirus. This new split supersedes the old classification of antigenic groups 1, 2, 3, and 4. The complete species list that was proposed by the International Committee on the *Taxonomy of Viruses (ICTV)*, which also included the genus that was found in birds. Alpha- and beta CoVs infect both humans and domestic animals, whereas gamma- and delta CoVs are primarily linked with avian hosts, even though they have been found in marine mammal species and in several Asian predators. Coronaviruses, also known as CoVs, are members of a broad family of viruses that are enclosed and have a genome consisting of a single strand of RNA. These viruses are always present in mammals and birds and pose a threat to humans as well as other animals, including livestock. The *Coronaviruses (CoVs)* that are carried by avian species are classified into the genera gamma- and delta coronaviruses respectively. The avian coronavirus is the most well-known member of the gamma-CoV family. Avian coronavirus is the technical term for the infectious bronchitis viruses (IBVs) that can be found in chickens and other domestic birds such as turkeys, guinea hens, and quails. These IBVs can also infect humans. IBVs have also been discovered in wild birds that were reported to be in good health, which suggests that wild birds could act as the vector between domestic and wild birds. In addition, other coronaviruses besides IBVs have been identified in wild birds, which suggests that these creatures play a significant role in the epidemiology of other *gamma CoVs and delta CoVs.* Representative alpha-coronaviruses include human coronavirus NL63 (HCoV-NL63), porcine transmissible gastroenteritis coronavirus (TGEV), PEDV and porcine Coronavirus (PRCV). Representative beta-coronaviruses include SARS-CoV, MERS-CoV, bat coronavirus HKU4, mouse hepatitis coronavirus (MHV), bovine coronavirus (BCoV) and human coronavirus OC43. Gamma representing delta - coronaviruses include bronchitis coronavirus (IBV) and porcine delta-coronavirus (PdCV) (5).

Additionally, the spike is a critical factor for viral load and tissue tropism and is the deep inducer of immune responses (6). Therefore, it appears that the link between SARS-CoV and respiratory epithelia plays a vital role in the genes of SARS. Cellular receptors are linked to other coronaviruses associated with human disease. Recently, angiotensin-converting enzyme 2 (ACE2) has been identified as the receptor for both SARS-CoV (7) and NSP3.The multi-domain non-structural protein 3 (Nsp3), which has an average molecular mass of about 200 kD, is the largest protein that the coronavirus (CoV) genome can encode. Nsp3 is an essential part of the replication/transcription complex. It is made up of several domains, whose organization differs amongst CoV genera due to domain duplication or absence. The ubiquitin-like domain 1 (Ubl1), the Glu-rich acidic domain (also known as the “hypervariable region”), a macrodomain (also known as the “X domain”), the papain-like protease 2 (PL2pro), and the Nsp3 ectodomain (3Ecto) are the eight Nsp3 domains that are present in all known CoVs. Additionally, the TM1 and TM2 transmembrane domains are present in all CoVs. Nuclear magnetic resonance (NMR) spectroscopy and/or X-ray crystallography have both been used to examine the three-dimensional structures of domains in the N-terminal two thirds of Nsp3 since the outbreaks of the Middle East Respiratory Syndrome coronavirus (MERS-CoV) in 2012 and the severe acute respiratory syndrome coronavirus (SARS-CoV) in 2003.

The rate of RNA virus mutations aids in viral adaptability by striking a balance between genomic variety and the integrity of genetic information. The biological characterisation of viral mutations can offer priceless insights for analysing immune evasion, pathogenesis, and viral treatment resistance related pathways. Studies on viral mutations are also important for developing novel vaccines, antiviral medications, and diagnostic tests. The viral enzymes that replicate the nucleic acids are responsible for the viral genome’s mutagenesis process, which is regulated by post-replicative nucleic acid repair and/or proofreading capabilities with little to none. Host enzymes, spontaneous nucleic acid damage brought on by physical and chemical mutagens, recombination events, and specific genetic components are other processes that cause mutations. Other variables, such as those that affect the template sequence and structure involved in viral replication, can influence the rate of mutation. Multi-domain proteins called RNA-dependent RNA polymerases (RdRps) can catalyse the creation of phosphodiester linkages between ribonucleotides in the presence of a divalent metal ion. With certain exceptions, such as the Nidovirales order (to which the Coronavirus genus belongs), which stands out for having the biggest RNA genomes, RNA polymerase lacks proofreading capabilities in most viruses. Coronavirus nsp3-RdRp plays a vital role in replication Non-structural proteins (nsps), which are created as byproducts of the cleavage of viral polyproteins to promote virus replication and transcription, control the sophisticated machinery used by nidoviruses to synthesize RNA (8). ACE2 is a membrane-bound aminopeptidase expressed in vascular endothelial, renal, and cardiovascular tissue and epithelia of the small intestine and testicles (9). The extracellular region ACE2 component, which includes first-helix and lysine 353 and adjacent remnants of the N-terminus of alpha-helix (sheet-5) interacts closely with the SARS-CoV S glycoprotein receptor binding compound (10). Similarly, SARS-CoV nsp3 is a major protein of 1922 (11) amino acids, thought to have at least seven domains: (1) N-terminal Glu-rich acidic (AD) domain; (2) X domain (XD) with poly binding (ADP-ribose) and SUD domain (SARS-CoV Unique Domain, entries not found in any other coronavirus to date) directly related to oligo (G) - strands (7, 12) a papain-like protease (PLP2), recently shown to show the function of removing ubiquitin (8, 13); an unknown domain that may extend the papain-like protease domain, called PLnc’s Papain-Like noncanonical domain (9); transmembrane domain (14) corresponding to the N-terminal of the Y-domain; and (10) the remainder of the Y domain, the abbreviation “Y domain” will be used for this part of this study. The transmembrane domains that distribute most of the nsp3, nsp4 and nsp6 are considered compact/compact (RTC) compact devices. Current advances in the field of vacciome (15–17) and the availability of various epitope drug design tools have greatly enhanced research into the development of potential vaccine candidates (18–22).

Epitope-based vaccine design is one of the reliable, accurate, and fast way to design vaccines against toxic viruses. Therefore, this study aimed to sign up a multi-epitope subunit vaccine against SARS-CoV-2. Here, we used three antigenic proteins (spike protein, nucleocapsid protein, and membrane protein) from SARS-CoV-2 to design B and T-cell epitopes in them. Similarly, HTL epitope prediction was performed, and a final vaccine composed of multiple epitopes was developed. In addition, we used a molecular entry method followed by a strong thermodynamics print, an *in-silico* expression profile, and an agent-based modeling device to ensure the stability, speech, and response of antibodies irritated by the last vaccine.

## Material and methods

### Virulence protein collection

**Figure 1:**
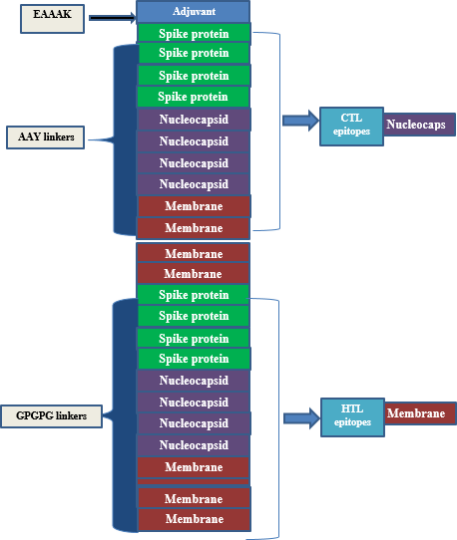
Schematic diagram to construct vaccine against SARS-COV-2.

Proteins are vital for the development of vaccines; therefore, we have selected three toxic proteins against Covid-19, including spike protein, nucleocapsid protein, and membrane protein, according to their role in covid-19 respiratory diseases. The workflow of this scientific study is shown in **Figure 1**.

### Predictions of CTL (MHC-I binding) epitope

Cytotoxic T-lymphocytes consist of CD8 and a subset of T cells that respond to the killing of cells charged by infra cellular fungus infections, viruses, bacteria, or protozoan. Similarly, CTL epitopes (MHC-I) are predicted in three negative covid-19 proteins (Spike protein, nucleocapsid protein, and membrane protein) by the online tool NetCTL1.2 (http://www.cbs.dtu.dk/services/NetCTL/) (23). Based on C-terminal cleavage values, transport-related proteins, and MHC binding points- I, this server predicts 9-mer protein epitopes. Then neural implantation system was followed by a C terminal cleavage server and binding peptides in MHC-I. The weight matrix obtained optimal mobility efficiency. Epitopes predicted at 0.75 (by default parameters) and reduced> E.

### Prediction of HTL (MHC-II binding) epitopes

Helper cells (T-lymphocyte) are the critical player in both humoral and cell-mediated immune responses. Therefore, HTL receptor-specific epitopes were possibly an essential part of the prophylactic and immunotherapeutic vaccine. In this study, for the selected three virulent proteins of covid-19, including Spike protein, nucleocapsid protein, and membrane protein, the MHC-II binding-epitopes (HTL) were predicted using a web tool IEDB MHC-II (http://tools.iedb.org/mhcii/). In this sense, we used MHC alleles, including HLA-DRB1*01:01, HLA-DRB3*01:01, HLA-DRB4*01:01, and HLA-DRB5*01:01 and IEBD recommended 2.22 predicted method to detect 15-mer length epitopes. This server defines the affinity of peptides towards the MHC-II based on the lower percentile ranked in **Table 1**.

**Table 1:** Helper-T-Lymphocytes epitopes are given in the table along with their scores predicted by IEDB MHC class II serve.

Alleles	Start	End	Peptide sequence	Method	Percentile rank
HLA-DRB1*01:01	513	527	LSFELLHAPATVCGP	Consensus (comb.lib./smm/nn)	0.03
HLA-DRB1*01:01	512	526	VLSFELLHAPATVCG	Consensus (comb.lib./smm/nn)	0.03
HLA-DRB1*01:01	511	525	VVLSFELLHAPATVC	Consensus (comb.lib./smm/nn)	0.03
HLA-DRB1*01:01	514	528	SFELLHAPATVCGPK	Consensus (comb.lib./smm/nn)	0.09
HLA-DRB4*01:01	218	232	TALALLLLDRLNQLE	Consensus (comb.lib./smm/nn)	0.66
HLA-DRB5*01:01	84	98	QIGYYRRATRRVRGG	Consensus (comb.lib./smm/nn)	0.67
HLA-DRB4*01:01	217	231	ETALALLLLDRLNQL	Consensus (comb.lib./smm/nn)	0.76
HLA-DRB5*01:01	83	97	DQIGYYRRATRRVRG	Consensus (smm/nn/sturniolo)	0.77
HLA-DRB1*01:01	144	158	IRGHLRMAGHSLGRC	Consensus (comb.lib./smm/nn)	0.19
HLA-DRB1*01:01	143	157	IIRGHLRMAGHSLGR	Consensus (comb.lib./smm/nn)	0.27
HLA-DRB1*01:01	145	159	RGHLRMAGHSLGRCD	Consensus (comb.lib./smm/nn)	0.31
HLA-DRB4*01:01	42	56	NRFLYIIKLVFLWLL	Consensus (comb.lib./smm/nn)	0.39

### Prediction of B-cell epitopes

B-cells are a chief player in the defense system of epitopes associated with the B-cell receptor, which plays a key role in developing antibody vaccines following antibody production. Therefore, to predict specific B-cell epitopes, the ABCPREDS (http://crdd.osdd.net/raghava/abcpred/) server has been used (24). ABCPRED is primarily responsible for protecting the immune system, which, in turn, provides long-term immunity. To predict B-cell linear epitopes, ABCPRED used a neural implant network based on a repetitive neural network (machine-based process) using patterns of limited length to predict 20-mer B-cell epitopes. Dissolved B cell epitopes are predicted by web server Ellipro (http://tools.iedb.org/ellipro/). The ElliPro suite uses fossil fusion algorithm and Thornton technology to predict B cells (conformational epitopes). This server mainly uses Jsmol to generate 3D links for server-predicted epitopes (25). Similarly, available data are available and confirm the immunodeficiency of a sub-unit vaccine designed.

### Composition of multi-epitope subunit vaccine

A synthetic vaccine of 386 amino acid residues was eventually developed with 12 HTL and 12 CTL epitopes, based on its high binding and non-allergenic properties naturally. To create a multi-epitope-subunit vaccine, the EAAAK connector was bound as an adjuvant with intra-CTL and Intra-HTL epitopes compiled by AAY and GPGPG linker, respectively. Eventually, vaccine formulation was achieved with adjuvant, linker, CTL, and HTL epitopes from N-terminal to C-terminal, respectively. These links are vital for two reasons; links effectively block the formation of neo-epitopes (junctional epitopes) and improve epitope presentation. The linkers such as AAY, EAAAK, and GPGPG are utilized rather frequently in the process of developing vaccines. Linkers being utilized so that the epitopes could be combined, and an adjuvant being incorporated into the procedure so that the immune response of the host could be stimulated. The VaxiJen 2.0 web server was used once more to do additional research into the antigenicity and allergenicity of the construct.

### Allergies to antigenicity treatment

Allergies and overuse of the immune system are experienced in the past, usually harmless substances that can cause skin rashes, rashes, inflammation of the mucous membranes, and sneezing. Allergies to these predictable components of the vaccine were determined using the online tool AllerTOP (https://www.ddg-pharmfac.net/AllerTOP/), and it found that the vaccine protein is anti-inflammatory and safe for human use. The vaccine prescribed to the person in charge should have a great immune system that can trigger a high immune response, leading to the formation of memory cells against pathogenic epitopes. To determine the immunodeficiency of a synthetic vaccine, two online servers including Vaxijen2.0 (http://www.ddg-pharmfac.net/vaxijen/VaxiJen/VaxiJen.html) (26) and AntigenPro (http://scratch.proteomics.ics.uci.edu/) were used by entering a build-up tree sequence as a query.

### Physiochemical properties

The antibodies of the predicted vaccine were determined with the ProtParam server (https://web.expasy.org/protparam/) and tested with seven parameters. This server determines the composition of accurate amino acids, an indicator of instability, aliphatic points, *in vivo* half-life, molecular weight, *in vitro* half-life, Grand Average of Hydropathicity (GRAVY), and pI theory (27).

### Secondary structure modeling

The secondary structure was predicted according to the vaccine sequence by the ‘PSIPRED’ web server (http://bioinf.cs.ucl.ac.uk/psipred/). In addition, this tool can be used for internal networks (feed-forward) to process Position Specific Scoring Matrix (PSSM) predictive properties for secondary properties using the PSIPRED (28).

#### 3D structure assumptions, refinement, and validation

In the 3D architecture prediction of vaccine formulation, the freely available web server Robetta (https://robetta.bakerlab.org/) has been used (29). This server is constantly updated with CAMEO and a visualization of the UCSF camera tool used. With the analysis of the predicted model, the Galaxy Refine online tool (http://galaxy.seoklab.org/) for continuous development (30). Similarly, the server uses the CASP10 command to upgrade the 3D query status. Alternatively, the side chains of proteins also rebuild through the CASP10 process followed by reloading. and using 3D architecture simulation for continuous relaxation. Possibly, the refinement of the Galaxy was used for the continuous development of 3D editors in terms of world-class and structural quality (30). For power reduction and structural adjustment, UCSC camera software was used. To validate the structure and test, ensure that 3D is used to analyze the coherence of the atomic (3D) model in its amino acid sequence (31), which is incorrect to test the arrangement of different atoms in the protein model and rampage tools used.

#### Codon efficacy and *in-silico* expression vaccine

The reverse translation and optimization of the *Java Codon Adaptation Tool (*Jcat**)** have been used, to obtain a high-level expression in *E. coli*. Jcat calculates CAI scores and GC content in question in order to achieve the highest quote. In the Prokaryotic ribosome, the termination and autonomy of the binding sites are selected. Similarly, the Ndel and Xhol limitation sites are set to follow the translated sequence. Finally, the prepared vaccine was combined with pET-32a + MEV plasmid with a snap-gene software package.

#### Molecular docking of vaccine with ACE2 and NSP3

Chemical insertion is a calculation method, which can predict the desired shape of the ligand molecule in the receptor molecule in its complex form. It also calculates the binding bond between the two molecules (ligand and receptor) in the terms of goal-scoring activities. Vaccine molecules made ACE2 and NSP3 proteins, directly affected by covid-19 was docked *via* the Cluspro online server (https://cluspro.org/login.php) (32).

#### Molecular dynamics simulation

The Desmond program of Schrödinger software 2021-2 (Schrödinger, LLC, New York, NY, USA) (33) with the OPLS4 (Optimized Potentials for Liquid Simulation) (34) force field was used for MD simulation to analyze protein conformational changes. A simulated triclinic periodic boundary box with a 10 extension in each direction was used to solve the GluN2B protein structures, and an explicit solvation model (Monte-Carlo equilibrated SPC) was used for each system. Lennard-Jones interactions (cut-off = 10) and the SHAKE algorithm governed covalent bond mobility (35). 0.15 M Na+Cl was added to neutralize the system during solvation. At 300 K and 1 bar pressure, the NPT ensemble class reduced the energy of protein models until a gradient threshold of 25 kcal/mol was reached. Each system had a single MD run, and all simulated trajectories were re-covered in 20 ps. The Particle Mesh Ewald (PME) method was utilized to compute long-range coulombic interactions, and the RESPA integrator (36) was used to govern all covalent bonds associated with hydrogen atoms, with an inner time step of 2 fs. For short-range electrostatic interactions, 9.0 was chosen; for long-range van der Waals (VDW), uniform density was employed. At 300 K and 1 atmosphere, a Nosé–Hoover thermostat (37) with a 12-ps relaxation time was used. The Martyna–Tobias–Klein barostat approach (38) with a 12 ps relaxation length was used. The molecular simulation was performed at 1 atm pressure and 300** K** temperature for a 100 ns NPT production run under the OPLS4 force field.Two post molecular dynamics simulation analysis i.e., RMSD and RMSF was performed for all the system.

### Imitation of MD simulation

**Figure 2:**
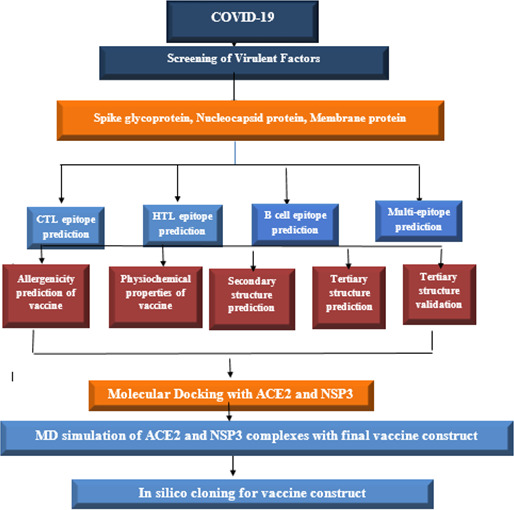
The structural arrangement of the final vaccine candidate constructed from CTL and HTL epitopes using different linkers.

The dynamics of a molecule were performed using Desmond. Desmond is a software developed at D.E. Shaw research, which analyzes the fastest mimicry of cells of biological processes in normal computer clusters. Ten cut cutoff radius of non-bond interaction is considered. Transmission analysis of ten nanoseconds trajectories was performed with PTRAJ52 and CPPTRAJ. Two statistical parameters are calculated RMSD and RMSF for all systems (**Figure 2**).

## Results

### Covid-19 protein sequence collection

In 2019, a new infectious illness caused by a coronavirus has emerged and is rapidly spreading over the globe. Severe acute respiratory syndrome coronavirus 2 is a new coronavirus that causes this condition (SARS-CoV-2). The S1 and S2 subunits make up the SARS-CoV-2 spike (S) protein, which is essential for receptor identification and cell membrane fusion. By generating a six-helical bundle *via* the two-heptad repeat domain, the S2 subunit enables viral cell membrane fusion, whereas the S1 subunit has a receptor-binding domain that identifies and attaches to the host receptor angiotensin-converting enzyme 2. The sequence of covid-19 protein spike proteins is composed of 1273 amino acids weighing 141,178 (Da), while the nucleocapsid protein contains 422 amino acids counting 46,025 (Da) and membrane protein containing 221 amino acids mass 25,061 (Da) retrieved from UniProt (https://www.uniprot.org/) database.

### Antigenicity of toxic proteins Covid-19

It is possible to have access to the antigenicity of a variety of covid-19 proteins by logging onto the web server Vaxijen2.0 (http://www.ddg-pharmfac.net/vaxijen/VaxiJen/VaxiJen.html). This server is located at: http://www.ddg-pharmfac.net/vaxijen/VaxiJen/VaxiJen.html. According to the research, these proteins are the ones that are responsible for carrying the antigen characteristics in a school that is continuously formed by a spike feeder, Nucleocapsid, and protein membranes (0.47, 0.48, and 0.59, respectively). In a similar fashion, the points for antigenicity potential reflect the anti-genic status of the selected sequence (proteins) that were used in the creation of a sub-unit vaccination. This was done to create a more effective vaccination.

### Prediction of CTL and HTT epitopes

Epitope prediction algorithms, with extensive allelic coverage, have recently been created, verified, and shown to be reliable techniques for predicting the binding affinities of peptides to MHC molecules. The identification of T-cell epitopes produced from the vaccine virus confirmed the efficacy and usefulness of computational in silico analysis for broadly finding CD8 + T-cell epitopes (39). 49 CTL epitopes were discovered in that ground-breaking study, and it was later shown that they accounted for 95% of the whole CD8 response to the Influenza viruses. Since then, several research have employed in silico epitope identification techniques to locate potential epitopes for inclusion in carefully crafted multivalent vaccinations meant to guard against diseases brought on by a variety of viral and bacterial pathogens. For instance, research has demonstrated that vaccinations based on epitopes can successfully elicit immune responses that are protective against a variety of diseases, including the influenza and HIV viruses. Strong CTL responses have been seen following epitope delivery by DNA vaccines or DNA-launched nanoparticle vaccinations.

The CTL epitopes were predicted using NetCTL, which estimated 58 CTL epitopes with a length of 9mer length. In all, only 12 epitopes were selected based on different terms of the binding effects of MHC and the non-allergenic nature of these three potent proteins responsible for the design of the vaccine. HTL epitopes predicted that MHC alleles HLA-DRB1 * 01: 01, HLA-DRB3 * 01: 01, HLA-DRB4 * 01: 01, and HLA-DRB5 * 01: 01 using a web tool binding to IEBD MHC-II continuously elevated 12 HTL epitopes 15 to 15 ounces from each harmful protein in different areas based on the lowest level of percentile (**Table 1**).

### Composition of multi-epitopes vaccine

The final vaccine is made up of 386 and 12 amino acids, which were selected because of their capacity to bind (they are CTL and HTL epitopes), the fact that they are non-allergenic, and the fact that they contain antigenic qualities. In addition to this, the amino acids were selected because of the ease with which they could be produced (**Table 2**). In addition to this, both the GPGPG linkage and the AAY linkage were incorporated into the process of developing the comprehensive multi-epitope vaccine. The CTL epitopes were included into the manufacturing process of the adjuvant in a manner that is analogous to the one described in the previous paragraph. This was accomplished through the utilization of the EAAAK link. GPGPG and AAYlinkers were able to successfully generate an attachment of HTL and CTL epitopes adjuvant to the N-terminus side of the anti-corrosion drug by combining their efforts. In order for this goal to be accomplished, the two linkers that were being utilized were consolidated into a single one. In addition to this, a piece of software known as BLAST was utilized in order to incorporate newly anticipated epitopes into the investigation. This was done to avoid using homologous epitopes along with different kinds of human proteins (**Figure 2** and **Table 2**).

**Table 2:** CTL and HTL epitopes were used to construct the final vaccine structure based on combined score.

Sequence	Combine score	Type of epitope
WTAGAAAYY	3.11	CTL epitope
TSNQVAVLY	3.08	CTL epitope
CVADYSVLY	2.58	CTL epitope
KTSVDCTMY	2.38	CTL epitope
LSPRWYFYY	2.34	CTL epitope
GTTLPKGFY	1.68	CTL epitope
LLNKHIDAY	1.39	CTL epitope
NTASWFTAL	0.95	CTL epitope
GTDSGFAAY	3.43	CTL epitope
YSNRNRFLY	2.73	CTL epitope
VATSRTLSY	1.46	CTL epitope
WIMLLQFAY	1.16	CTL epitope
LSFELLHAPATVCGP	0.03	HTL epitope
VLSFELLHAPATVCG	0.03	HTL epitope
VVLSFELLHAPATVC	0.03	HTL epitope
SFELLHAPATVCGPK	0.09	HTL epitope
IRGHLRMAGHSLGRC	0.19	HTL epitope
IIRGHLRMAGHSLGR	0.27	HTL epitope
RGHLRMAGHSLGRCD	0.31	HTL epitope
NRFLYIIKLVFLWLL	0.39	HTL epitope
TALALLLLDRLNQLE	0.66	HTL epitope
QIGYYRRATRRVRGG	0.67	HTL epitope
ETALALLLLDRLNQL	0.76	HTL epitope
DQIGYYRRATRRVRG	0.77	HTL epitope

### Predictions of B cell epitopes

**Figure 3:**
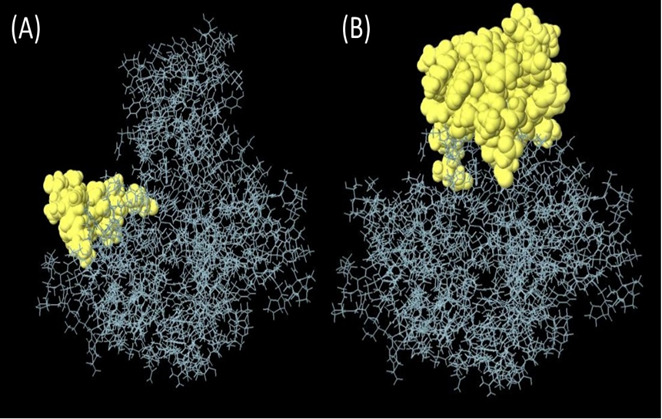
**(A)** Linear epitopes predicted by ABCpred and **(B)** discontinuous (conformational) epitopes predicted by ellipro visualized in jsmol, yellow part showed predicted epitope.

B cell epitopes that have been predicted with the use of a process referred to as ABCpred, which is a complex computer algorithm. The utilization of this approach allowed to produce these forecasts. This strategy was utilized throughout the course of the investigation so that we could arrive at these conclusions. Using this method, 20-mer epitopes are selected for further study depending on the number of exceptional epitope sites that can be discovered in each of the areas in which they are located. This is done to maximize the amount of information that can be gleaned from the study. This assists in reducing the number of possible candidates that need to be investigated. The National Center for Biotechnology Information (NCBI) is the organization that is responsible for the creation of this methodology. On the other hand, we utilized continuous epitopes that totaled 55 amino acids in length when combined. These are the ones that, in our opinion, stand out as being particularly remarkable. Following that, these epitopes were disassembled, and then reassembled into a three-dimensional pharmacological model with a probability of around 0.618. In a method that is somewhat like this, the epitopes are designated by the yellow zone, whereas most of the polyprotein is shown by the gray region (**Figure 3**).

### Characterization of physiochemical properties and secondary structure prediction

The result also showed that the last vaccine is non-allergic with 0.32 points in the 0.4 automatic thresholds calculated using the Vaxigen 2.0 sever (21). Since causal microorganisms no longer need to be cultivated, the creation of vaccines in the post-genomic age frequently starts with an in-silico analysis of genome information to identify the most likely protective antigens. Despite the approach’s obvious benefits, like speed and cost effectiveness, its success still depends on how well the antigen is predicted. Most methods identify antigens by sequence alignment. This is a problem for several reasons. Even while certain proteins may have comparable structures and biological characteristics, some proteins lack evident sequence similarities. A sequence’s antigenicity may be encoded in a deceptive way that made it impossible to directly identify by sequence alignment. The absence of similarity to antigens of known provenance will prevent the development of truly novel antigens. We provide a new alignment-free method for antigen prediction to get over the drawbacks of alignment-dependent methods. Models for the prediction of entire protein antigenicity were developed using protein datasets. 100 recognized antigens and 100 non-antigens made up each group. Internal leave-one-out cross-validation and external validation utilizing test sets were used to evaluate the resulting models. The stability of the distinction between antigens and non-antigens was evaluated using an additional five training sets for each class of antigens. Both validations of the models showed good performance, with prediction accuracy ranging from 70% to 82.85%. The models were put into practice on a server that we’ve named VaxiJen2.0.

**Figure 4:**
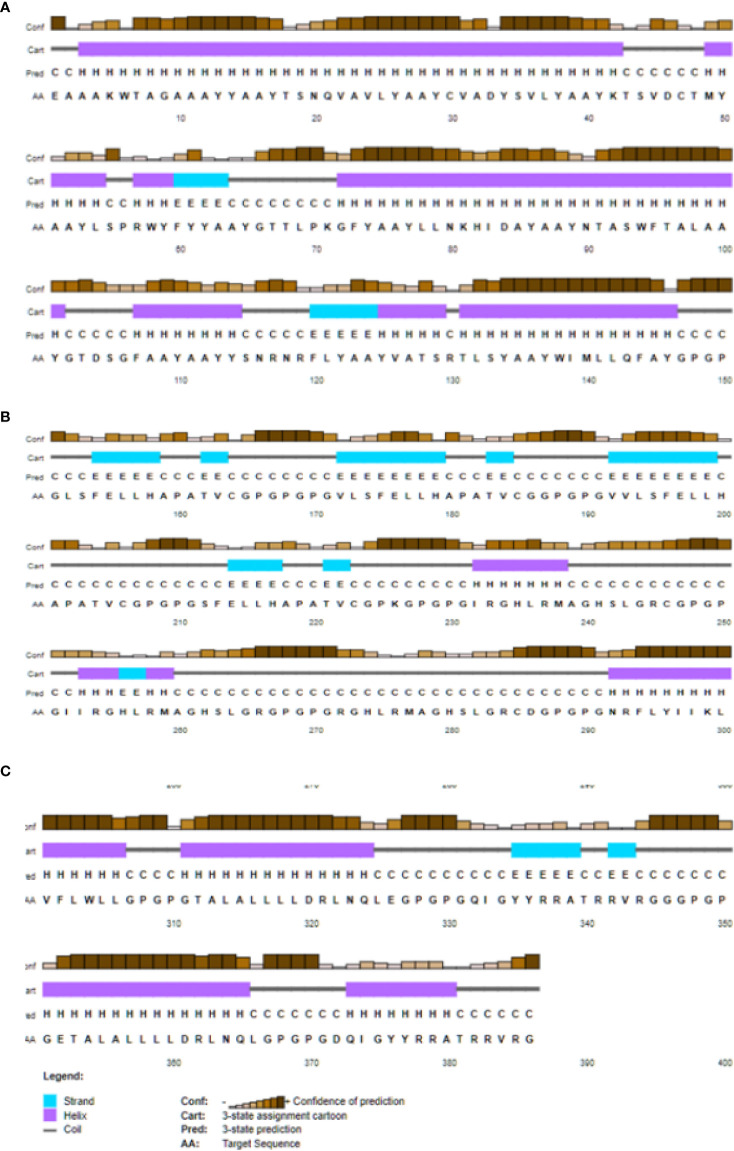
PSIPRED web tool predicted secondary structure elements Early predictions cantered on helix- or sheet-forming amino acids and free energy. 60% accurate residue (helix/sheet/coil) prediction. Multiple sequence alignment boosted accuracy to 80%; knowing amino acid distribution at a site (and in its surroundings) sharpens understanding of structural patterns there. Random coils may form in glycine-containing proteins.95% of homologous proteins have helix-favouring amino acids. Hydrophobicity may imply a -helix-compatible pattern of residue solvent accessibility. The secondary **(A)** represent the prediction model consisted of 47.2% α-helix, **(B)** 42.0% β-sheet, and **(C)** and 10.0% coils.

At the same time, the weight of the vaccine is as high as 40825.88, which is likely to favor the weakness of the last vaccine. Similarly, the theoretical pI is to be approximately 9.54. The average life expectancy in mammalian reticulocytes was 1hours, *in vitro*, while 30 min in yeast and 10 hours *E. coli in vivo*. With an extinction coefficient of 7568 and an instability rating of 33.29, it’s clear that this medicine is in a relatively stable state. Also, the highest value of the aliphatic index was seen (82.85), while the lowest value of the sub-unit vaccine, hydropathicity (GRAVY), was seen (0.046) and immunosuppressive medicine. The second structure for the construction of the vaccine was predicted using a cartoon PSIPRED image and a CFSSP online server for the construction of the secondary structure. The secondary prediction model consisted of 47.2% α-helix, 42.0% β-sheet, and 10.0% coils (**Figure 4**).

### Tertiary structures prediction, refinement, and validation

**Figure 5:**
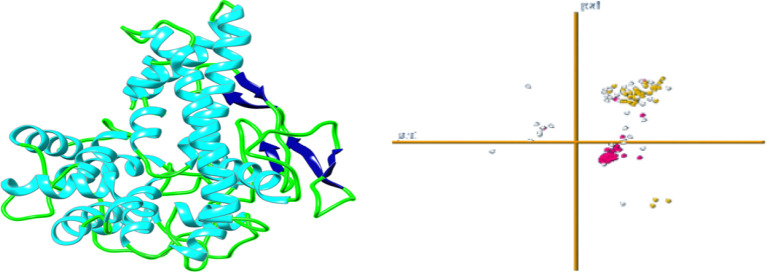
3D predicted tertiary structure of vaccine construct.

**Figure 6:**
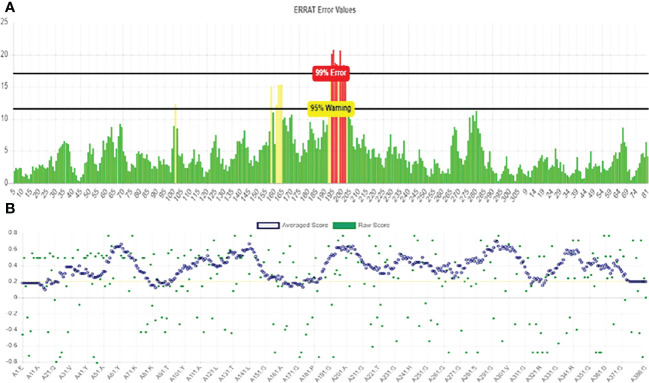
Verification of the vaccine construct model using **(A)** ERRAT plot for vaccine construct model and **(B)** Verify 3D results.

**Figure 7:**
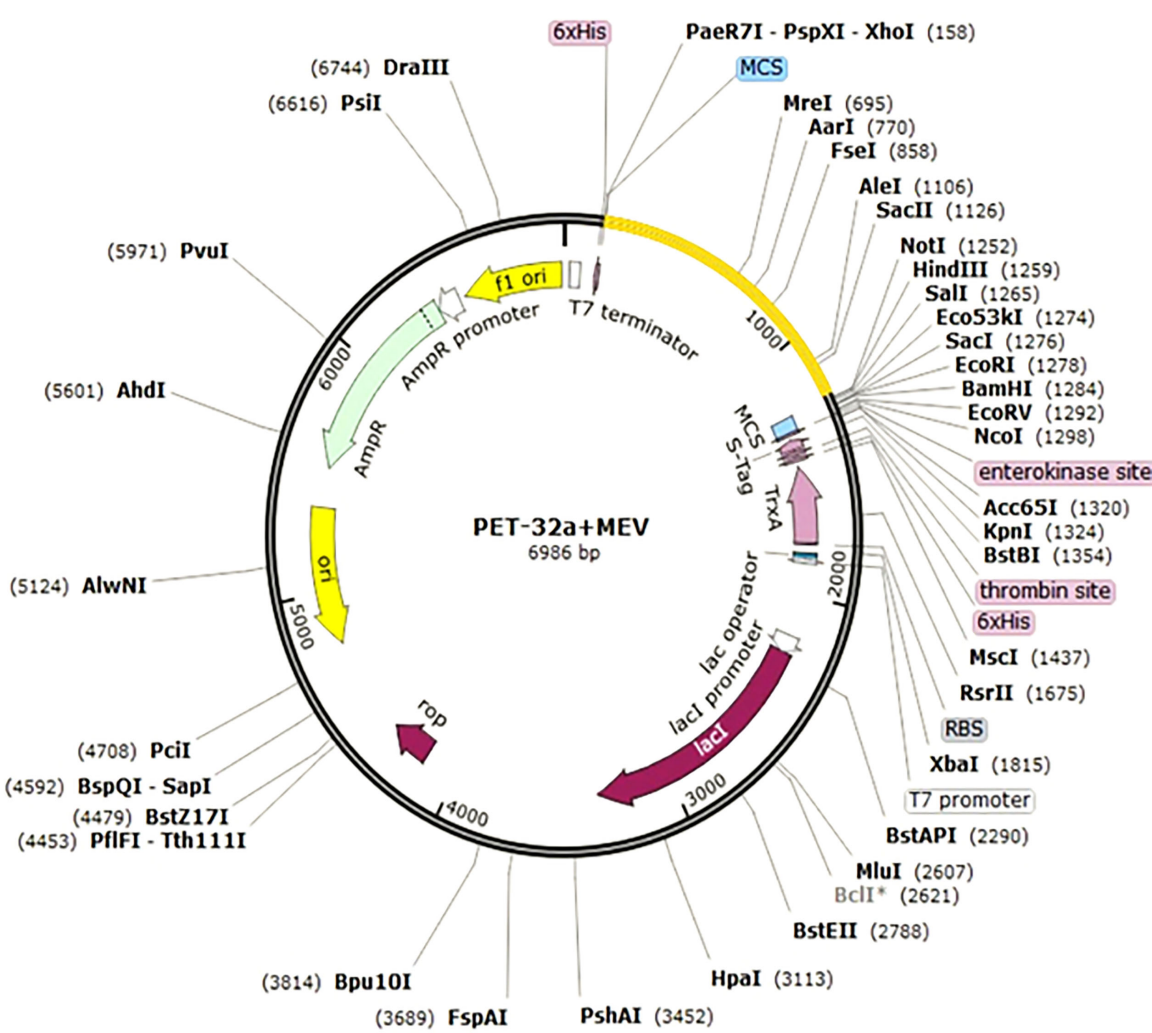
In silico cloning for adapted vaccine sequence into pET32a+MEV vector. Different epitopes of three proteins and merged it one sequence and then out sequence show this vector image. Our sequence is probably attached every clone vector.

Robetta’s web server used an abinitio method model to generate the final multi-epitope vaccine design’s three-dimensional structure. This was done to ensure that the vaccine would be effective. This structure was erected specifically for the administration of vaccinations. This server generates forecasts for the five different built-in policy versions that have the best chance of being the most successful overall. The z-score value that the UCSF camera determined to be the most accurate may be used to select the first model as the one that would be utilized to represent the data. This would ensure that the most accurate picture possible is produced. This option is available for selection. The strands, which are made up of black helixes, are represented by the color. The Green represents the coils, while blue cyan represents the strands that are made up of the black helixes (**Figure 5**). Similarly, the Galaxy Refine server was tracked to further improve the selected model-1, tested with GDT-HA (0.9916), RMSD (0.262), Ramachandran strategy (97.4), MolProbity (1.736)), and bad rotamers (0.7) and school conflict (12.8). Similarly, power is reduced with UCSF Chimera software and the structure was also tested to check the quality with the ERRAT web tool (**Figure 6**), which indicates a standard 95.24 standard. The vertical axis represents points, while the horizontal axis represents the protein residue. (**Figures 6A, B**). The 3D architecture validation revealed that 87.82% of the fossils had an estimated 3D-1D scale> = 0.2 (**Figure 7**). The **Figure 5** **is** analysis of the Ramachandran Plan also showed that 97.4% of the remains were in the best region (> 90%), of all, 0.8% in the permitted area, and 0.0% in the outdoor area. The Ramachandran plot represents peptide torsional angles (phi and psi) in two dimensions. the torsion angles N(i-1),C(i),Ca(i),N(i) and C(i),N(i),C(i+1). G. N. Ramachandran et al. produced the plot by putting on the x-axis and on the y-axis in 1963. This graphic shows what torsional angle variants are possible. Each residue’s torsional angle determines the placement of its planar peptide bond in reference to that of its two neighbors. Steric hindrance prevents various angle combinations and residue conformations. Ramachandran plots reveal which torsional angles are permitted in peptide structure. Right: ribonuclease H’s Ramachandran plot.

#### Codon alteration and in silico cloning

Here, the Jcat server was used to quantify the expression of the multi-epitope *E. coli* vaccine (type K12). Satisfactory inputs used 1150 nucleotides in total. Similarly, codon production was performed, and the Codon Adaptation Index (CAI) is known as 0.94, while GC content is 59%. The clone length was 6986 kbp, and NdeI and XhoI block sites were created and integrated into the pET32a + Mev vector (**Figure 7**). The sequence of the target in the Clone is indicated in blue between the block areas. The targeted sequence is surrounded by 6-histidine residues on both sides, which will assist in purification purposes.

### Molecular docking of vaccine complex

The conclusion of the research on molecular docking that was carried out with ClusPro resulted in the construction of a total of thirty different kinds of structures. Because the binding energies of those 10 dock complexes were the lowest compared to the other thirty that might have been used, those ten were chosen as the best ones to use. In addition to those alternatives, we also considered several others. It was able to retrieve the dock complexes in the very best state that it would have been feasible for them to be in at the time of their retrieval. This was the very best state that it would have been possible for them to be in. They were in the absolute finest conceivable state that could have been achieved by them now. In a similar vein, protein-protein interaction complexes and the method by which they preferentially bind offer for a great engagement with the final vaccine. Even though the proteins ACE2 and NSP3 are part of these complexes, this is not an exhaustive list of the components that go into making up these complexes. This is since these complexes have limited connectivity capabilities, which means that they can only attach themselves to specific proteins. The reason for this is because these complexes have restricted connection capabilities (**Figure 1**).

### Interaction analysis of dock complex

**Figure 8:**
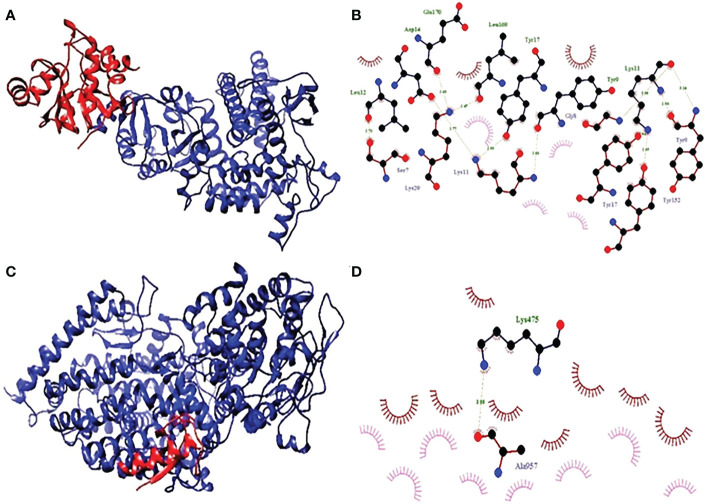
**(A)** Hydrophobicity and **(B)** interaction analysis of ACE3 with final vaccine construct. **(C)** Hydrophobicity and **(D)** interaction analysis of NSP3 with final vaccine construct.

The complex’s two-dimensional structure, which is made up of the protein and the ligand, as well as how these two elements interact with one another, are depicted in a diagram on the page. The website also includes a graphic that shows the interaction between the ligand and the protein (**Figure 8**). The statistical analysis’s results make it clearly clear that the receptor molecule and the peptide units interact on an atomic level. The results of the investigation directly led to the conclusion that was made. This conclusion may be deduced from the investigation’s findings. These conclusions were reached after considering the study’s findings, which allowed us to reach the ones mentioned previously in this paragraph. The Van der Waals force, ion contacts, and hydrogen bond formation are a few examples of these interactions, however they can take many different forms. One sort of contact is represented by the formation of hydrogen bonds

By connecting existing atoms with dashed lines, hydrogen bonding is indicated. Hydrogen from the protein itself is used to connect the remaining atoms. In a similar way to the preceding one, an arc is also used to represent hydrophobic interactions, where an arc represents protein residues. The ligand atoms connected to these connections, on the other hand, are the focus of the light speakers’ attention. Residues and receptors are both capable of developing interactions with one another. In addition to exhibiting different interactions, such as the interaction of hydrogen, ionic, and Vander Waals, residues and receptors also form π-π interactions.

### Molecular dynamic simulation analysis

**Figure 9:**
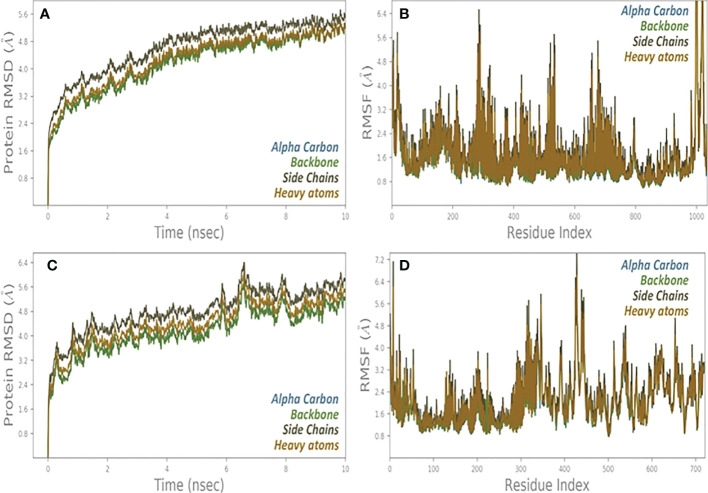
**(A)** RMSD and **(B)** RMSF analysis of MD simulation trajectories of vaccine complex with ACE2 receptor. **(C)** RMSD and **(D)** RMSF analysis of MD simulation trajectories of vaccine complex with NSP3 receptor.

The “Fluctuation plot” tab oversees building a collaborative 2D structure that correctly displays the residual output seen in all MD simulations. This structure depicts the residual output in the appropriate manner. This tab can be found in the centre of the window’s interface. Given that RMSF will take place later than the global position, it is reasonable to anticipate that there will be some variation in the value of this variable. A one-of-a-kind structure that is made up of a single chain is created as a result of the presence of several protein chains in a system. Monitoring the RMSD protein can offer information about the ordered structure of the protein across all of its mimics. This information can be useful in identifying potential problems. This information may be helpful in determining the likelihood of certain problems. When it comes to relatively small proteins like those that can be found all over the world, it is not a problem at all to switch up the order of the first three locations. A handy tool for reflecting local changes in the protein line is called the Rotated Root Factor, which is also commonly referred to as RMSF for its shorter form. It was determined that there were still some ACE2 and NSP3 receptors present because to the severity of the intermediate RMSF 2 that was discovered (**Figure 9**).

## Discussion

The scientific field of bioinformatics makes use of a wide range of computer technology for the purposes of predicting genetic factors, measuring texts of a certain kind, protein structures, and cell-based locations. In a similar manner, diseases and cell mutations are related with the non-symptomatic features of the proteins that are impacted by the mutation. One example of how this approach might be put into practice is the prediction of a viable pharmacological formulation based on proteins that are engaged in the pathophysiological process of a certain sickness. Research that is centered on a sequential order is still yielding profitable results as of this moment in time about the examination of the possibilities for covid therapy (22). This is because there is now an abundance of data and information that can be easily accessed on the proteomes and genomes of a wide range of viruses. Because of this, vaccinations are built up entirely of peptides, and the creation of these peptides may be helped along by a broad range of bioinformatics techniques (23). However, even though the concept of a vaccination based on an epitope is already in its early phases of research for a range of infectious illnesses, it is still challenging to reach the goal of developing a SARS-COV-2 epitope vaccine.

Research that is based on sequential steps is still producing fruitful results in terms of exploring the potential applications of covid treatment at the present time. This is due to the wealth of data and information that is now readily available on the proteomes and genomes of a wide variety of viruses. The production of vaccines, which are made up entirely of peptides, is possible with the support of a wide variety of various bioinformatics techniques. Nevertheless, even though the idea of a vaccination based on an epitope is now in its early phases of research for a variety of infectious diseases, the development of a SARS-COV-2 epitope vaccine is still difficult to achieve. In the current research, an attempt is made to create an in-silico epitope vaccine for SARS-CoV2 that, ideally, will be successful in treating both illnesses at the same time. This is the goal of the research. It considers this to be a standard modification, which may be compared to the distribution of a low-volume vaccination containing *E. coli* to a select group of people. Considering this fact, we made the decision to carry out this experiment using the vaccination method to evaluate the efficacy of the vaccine protein, as well as its stability and the presence of significant amounts of *E. coli*. Specifically, we wanted to find out whether the protein could withstand the presence of *E. coli* (K12 type). By far the most frequent host strains for pDNA propagation are E. coli K-12 strains (40). To select the best host strain for pDNA processing, depending on both the quality and quantity of supercoiling, the E. coli genotype is important (40).

The great majority of pDNA replications are carried out by *E. coli* K-12 strains, making them the most prevalent host strains (40). When looking for the best host strain for pDNA processing, it is critical to consider the genotype of the *E. coli* strain in question. When making this decision, you will need to consider both the level of supercoiling and its total quantity (40). In contrast, the use of complete agent-based studies examined the specific reaction when a drug sequence was introduced. The vaccine may be found to be non-allergenic, antigenic, safe, and can effectively control the coronavirus (24). By using clinical trials, it is still necessary to investigate the reliability and effectiveness of the vaccine. As such, this is mandatory to develop novel drugs in a very short time to find a way to control the possibility of progressively increasing diseases (25).

In addition, the physio-chemical properties of the vaccine were particularly evident in the use of the online prototype ProtParam, tested based on seven parameters, including half-life, aliphatic index and isoelectric point, molecular weight, instability index, *Grand Average of Hydropathicity* (GRAVY), and -coefficients of extinction. With an extinction coefficient of 7568 and an instability rating of 33.29, it’s clear that this medicine is in a relatively stable state. Also, the highest value of the aliphatic index was seen (82.85), while the lowest value of the sub-unit vaccine, hydropathicity GRAVY, was seen (0.046). The estimated value of the aliphatic index represents a subunit vaccine designed for the high value of the aliphatic index, the higher the height. At the same time, the negative GRAVY value of the subunit input drug represents the status of the hydrophobic vaccine. Overall, a vaccine is designed for immunogenic, thermostable, and hydrophobic (12). The predicted higher education structure of the vaccine (**Figure 5**) showed that all the formed lines were in the allowed range. Therefore, showed that the available model structure represented a high-level model. This finding is a pivotal result because a high percentage of the remains were in the preferred location. Similarly, the probability points further confirmed the immunogenic findings and the validity of the proposed vaccine (26, 41).

Docking shows the binding position of the connecting molecule towards the receivers. The 3D structure of the vaccine and the receptors considered to be receptor molecules is shown in (**Figure 8**). The results or suspensions of the vessels showed excellent interaction of the last vaccine composed with receptor proteins. Chain protein receptors ACE2 and NSP3 showed interactions with the multi-epitope vaccine, as can be seen in (**Figure 9**). These statistics have shown that both the recipient chains and the vaccine chains are represented here in different colors on arrival. By the interaction between vaccine peptide residues and the amino acids ACE2 and NSP3 receptor protein. The chain’s excellent binding location to the region that interacts with NSP3 and ACE2 suggests that it may be obstructing the interaction between the spike and ACE2 and blocking viral entrance into the host cells. It demonstrated a strong affinity for various SARS-CoV-2 structural and non-structural proteins (NSPs), which play a variety of roles in the life cycle of the virus. Significant binding of ACE2 with RdRp points to its function in preventing viral replication and, ultimately, virus proliferation. It demonstrated a strong affinity for various SARS-CoV-2 structural and non-structural proteins (NSPs), which play a variety of roles in the life cycle of the virus. Significant binding of ACE2 with RdRp points to its function in preventing viral replication and, ultimately, virus proliferation. The interaction that is depicted by LIGPLOT + is the interaction that is not only mediated by hydrophobic contacts but also by hydrogen bonds. This interaction takes place between two molecules. This is something that may be detected in the binding combination of active ACE2 residues and NSP3 receptors with multi-epitope immunization units that are compressed into a binding-protein packet. This is something that may be observed. Vaccines have the capacity to generate positive connections with ACE2 and NSP3 receptors, as demonstrated by the findings of this interaction analysis, which found that immunizations have this potential. The immunological response involves the participation of these receptors. This provides an explanation for why vaccinations have the potential to play a significant role in the case of covid-19. The time-dependent behavior of multi-epitope vaccines induced by ACE2 and NSP3 receptors modelled for 10-ns MD simulations to seek changes in the structures of both interacting particles adopted during simulation. The time-dependent behavior of multi-epitope vaccines induced by ACE2 and NSP3 receptors modelled for 10-ns MD simulations to seek changes in the structures of both interacting particles adopted during simulation. It was chief to understand enough changes to affect the binding of the construction on the fixed side. The epitope sequence was expressed in the body’s immune system, to inform and build up immune responses. In thus study, two statistical parameters calculate for this program including RMSD and RMSF (**Figure 9**). RMSD measured the innate distance between the atomic nuclei of a large protein. The RMSD structure shows the RMSD protein emergence (left of the Y-axis). All independent frames align to the spinal cord of the frame, and then the RMSD is calculated based on the choice of atoms.

## Conclusion

The COVID-19 epidemic is not only a health crisis, but also a global political, social, and economic crisis. The main goal of vaccine development is to develop a safe and effective vaccine that can reduce this fatal infection rate. The design of vacciome-based vaccines is relatively stable, reliable, fast, safe, expensive, and effective. We predicted epitopes and collaborated with them to develop a multidisciplinary epitope vaccine. Our evaluation was based on an analysis of the structure of the individual receiving the vaccine and the inclusion of cells and the study of MD simulations. The structures of ACE2 and NSP3 were very stable in their natural function, with a strong concentration of cells around ten ns. Our target candidate can stimulate the immune system and immune system by providing that these B and T-cells are selected in the final vaccine formulation. By doing it all together, according to physicochemical tests and immunological analysis and formulation, this vaccine can reduce the rate of infection (SARS-COV-2).

## Data availability statement

The original contributions presented in the study are included in the article/supplementary material. Further inquiries can be directed to the corresponding authors.

## Author contributions

Conceptualization, MSK, IMK and SUA; methodology, IMK and ZA; software, SN and MZK; validation, YL, MSK and IMK; formal analysis, IR; investigation, YL; resources, YL; data curation, MSK; writing-original draft preparation, MSK; writing-review and editing, YL; IMK; visualization, SN; supervision, YL; project administration, YL. All authors contributed to the article and approved the submitted version.

## References

[1] Chen N Zhou M Dong X Qu J Gong F Han Y. Epidemiological and clinical characteristics of 99 cases of 2019 novel coronavirus pneumonia in wuhan, China: a descriptive study. Lancet (2020) 395(10223):507–13. doi: 10.1016/S0140-6736(20)30211-7 PMC7135076 32007143

[2] Kumar N Singh A Grover S Kumari A Kumar Dhar P Chandra R. HHV-5 epitope: A potential vaccine candidate with high antigenicity and large coverage. J Biomolecular Structure Dynamics (2019) 37(8):2098–109. doi: 10.1080/07391102.2018.1477620 30044169

[3] Kumar N Sood D Chandra R. Design and optimization of a subunit vaccine targeting COVID-19 molecular shreds using an immunoinformatics framework. RSC Adv (2020) 10(59):35856–72. doi: 10.1039/D0RA06849G PMC9056885 35517103

[4] Zhao J Falcón A Zhou H Netland J Enjuanes L Pérez Breña P. Severe acute respiratory syndrome coronavirus protein 6 is required for optimal replication. J Virol (2009) 83(5):2368–73. doi: 10.1128/JVI.02371-08 PMC2643704 19091867

[5] Kumar N Sood D Sharma N Chandra R. Multiepitope subunit vaccine to evoke immune response against acute encephalitis. J Chem Inf Modeling (2020) 60(1):421–33. doi: 10.1021/acs.jcim.9b01051 31873008

[6] Liang JQ Fang S Yuan Q Huang M Chen RA Fung TS. N-linked glycosylation of the membrane protein ectodomain regulates infectious bronchitis virus-induced ER stress response, apoptosis and pathogenesis. Virology (2019) 531:48–56. doi: 10.1016/j.virol.2019.02.017 30852271 PMC7112112

[7] Branco ACCC Sato MN Alberca RW. The possible dual role of the ACE2 receptor in asthma and coronavirus (SARS-CoV2) infection. Front Cell infection Microbiol (2020) 10:550571. doi: 10.3389/fcimb.2020.550571 PMC7538685 33072624

[8] Tan YW Fung TS Shen H Huang M Liu DX. Coronavirus infectious bronchitis virus non-structural proteins 8 and 12 form stable complex independent of the non-translated regions of viral RNA and other viral proteins. Virology (2018) 513:75–84. doi: 10.1016/j.virol.2017.10.004 29035788 PMC7112110

[9] Santos RA Ferreira AJ Simões e Silva AC. Recent advances in the angiotensin-converting enzyme 2–angiotensin (1–7)–Mas axis. Exp Physiol (2008) 93(5):519–27. doi: 10.1113/expphysiol.2008.042002 18310257

[10] Jia HP Look DC Shi L Hickey M Pewe L Netland J. ACE2 receptor expression and severe acute respiratory syndrome coronavirus infection depend on differentiation of human airway epithelia. J Virol (2005) 79(23):14614–21. doi: 10.1128/JVI.79.23.14614-14621.2005 PMC1287568 16282461

[11] Imbert I Snijder EJ Dimitrova M Guillemot J-C Lécine P Canard B. The SARS-coronavirus PLnc domain of nsp3 as a replication/transcription scaffolding protein. Virus Res (2008) 133(2):136–48. doi: 10.1016/j.virusres.2007.11.017 PMC7114086 18255185

[12] Ahmad SU Kiani BH Abrar M Jan Z Zafar I. Ali Y. A comprehensive genomic study, mutation screening, phylogenetic and statistical analysis of SARS-CoV-2 and its variant omicron among different countries. J Infect Public Health (2022) 15(8):878–91. doi: 10.1016/j.jiph.2022.07.002 35839568 PMC9262654

[13] Nicholson B Leach CA Goldenberg SJ Francis DM Kodrasov MP Tian X. Characterization of ubiquitin and ubiquitin-like-protein isopeptidase activities. Protein Sci (2008) 17(6):1035–43. doi: 10.1110/ps.083450408 PMC2386736 18424514

[14] Kanjanahaluethai A Chen Z Jukneliene D Baker SC. Membrane topology of murine coronavirus replicase nonstructural protein 3. Virology (2007) 361(2):391–401. doi: 10.1016/j.virol.2006.12.009 17222884 PMC1925034

[15] Kumar N Admane N Kumari A Sood D Grover S Prajapati VK. Cytotoxic T-lymphocyte elicited vaccine against SARS-CoV-2 employing immunoinformatics framework. Sci Rep (2021) 11(1):7653. doi: 10.1038/s41598-021-86986-6 33828130 PMC8027208

[16] Agarwal D Zafar I Ahmad SU Kumar S Sundaray JK Rather MA. Structural, genomic information and computational analysis of emerging coronavirus (SARS-CoV-2). Bull Natl Res Cent. (2022) 46(1):1–16. doi: 10.1186/s42269-022-00861-6 PMC9199328 35729950

[17] Kumar N Sood D Chandra R. Vaccine formulation and optimization for human herpes virus-5 through an immunoinformatics framework. ACS Pharmacol Trans Sci (2020) 3(6):1318–29. doi: 10.1021/acsptsci.0c00139 PMC7737318 33344905

[18] Khan M Khan S Ali A Akbar H Sayaf AM Khan A. Immunoinformatics approaches to explore helicobacter pylori proteome (Virulence factors) to design b and T cell multi-epitope subunit vaccine. Sci Rep (2019) 9(1):1–13. doi: 10.1038/s41598-019-49354-z 31527719 PMC6746805

[19] Zafar I Iftikhar R Ahmad SU Rather MA. Genome wide identification, phylogeny, and synteny analysis of sox gene family in common carp (Cyprinus carpio). Biotechnol Rep (2021) 30:e00607. doi: 10.1016/j.btre.2021.e00607 PMC8076717 33936955

[20] Khan S Khan A Rehman AU Ahmad I Ullah S Khan AA. Immunoinformatics and structural vaccinology driven prediction of multi-epitope vaccine against mayaro virus and validation through in-silico expression. Infect Genet Evol (2019) 73:390–400. doi: 10.1016/j.meegid.2019.06.006 31173935

[21] Khan S Ali SS Zaheer I Saleem S Ziaullah Zaman N. Proteome-wide mapping and reverse vaccinology-based b and T cell multi-epitope subunit vaccine designing for immune response reinforcement against porphyromonas gingivalis. J Biomolecular Structure Dynamics (2022) 40(2):833–47. doi: 10.1080/07391102.2020.1819423 32928063

[22] Gul H Ali SS Saleem S Khan S Khan J Wadood A. Subtractive proteomics and immunoinformatics approaches to explore bartonella bacilliformis proteome (virulence factors) to design b and T cell multi-epitope subunit vaccine. Infect Genet Evol (2020) 85:104551. doi: 10.1016/j.meegid.2020.104551 32931955

[23] Larsen MV Lundegaard C Lamberth K Buus S Lund O Nielsen M. Large-Scale validation of methods for cytotoxic T-lymphocyte epitope prediction. BMC Bioinf (2007) 8(1):1–12. doi: 10.1186/1471-2105-8-424 PMC2194739 17973982

[24] Saha S Raghava GPS. Prediction of continuous b-cell epitopes in an antigen using recurrent neural network. Proteins: Structure Function Bioinf (2006) 65(1):40–8. doi: 10.1002/prot.21078 16894596

[25] Ponomarenko J Bui H-H Li W Fusseder N Bourne PE Sette A. ElliPro: a new structure-based tool for the prediction of antibody epitopes. BMC Bioinf (2008) 9(1):1–8. doi: 10.1186/1471-2105-9-514 PMC2607291 19055730

[26] Doytchinova IA Flower DR. VaxiJen: a server for prediction of protective antigens, tumour antigens and subunit vaccines. BMC Bioinf (2007) 8(1):1–7. doi: 10.1186/1471-2105-8-4 PMC1780059 17207271

[27] Bashir Z Ahmad SU Kiani BH Jan Z Khan N Khan U. Immunoinformatics approaches to explore B and T cell epitope-based vaccine designing for SARS-CoV-2 Virus. Pak J Pharm Sci (2021) 34. 34275860

[28] McGuffin LJ Bryson K Jones DT. The PSIPRED protein structure prediction server. Bioinformatics (2000) 16(4):404–5. doi: 10.1093/bioinformatics/16.4.404 10869041

[29] Kim DE Chivian D Baker D. Protein structure prediction and analysis using the robetta server. Nucleic Acids Res (2004) 32(suppl_2):W526–31. doi: 10.1093/nar/gkh468 PMC441606 15215442

[30] Heo L Park H Seok C. GalaxyRefine: Protein structure refinement driven by side-chain repacking. Nucleic Acids Res (2013) 41(W1):W384–8. doi: 10.1093/nar/gkt458 PMC3692086 23737448

[31] Eisenberg D Lüthy R Bowie J. VERIFY3D: assessment of protein models with three-dimensional profiles. Methods Enzymology (1997) 10:396–404. doi: 10.1016/S0076-6879(97)77022-8 9379925

[32] Kozakov D Hall DR Xia B Porter KA Padhorny D Yueh C. The ClusPro web server for protein–protein docking. Nat Protoc (2017) 12(2):255–78. doi: 10.1038/nprot.2016.169 PMC5540229 28079879

[33] Rani I Kalsi A Kaur G Sharma P Gupta S Gautam RK. Modern drug discovery applications for the identification of novel candidates for COVID-19 infections. Ann Med Surg (2022) 104125. doi: 10.1016/j.amsu.2022.104125 PMC9273307 35845863

[34] Doherty B Zhong X Gathiaka S Li B Acevedo O. Revisiting OPLS force field parameters for ionic liquid simulations. J Chem Theory Comput (2017) 13(12):6131–45. doi: 10.1021/acs.jctc.7b00520 29112809

[35] Kräutler V Van Gunsteren WF Hünenberger PH. A fast SHAKE algorithm to solve distance constraint equations for small molecules in molecular dynamics simulations. J Comput Chem (2001) 22(5):501–8. doi: 10.1002/1096-987X(20010415)22:5<501::AID-JCC1021>3.0.CO;2-V

[36] Tuckerman M Berne BJ Martyna GJ. Reversible multiple time scale molecular dynamics. J Chem Phys (1992) 97(3):1990–2001. doi: 10.1063/1.463137

[37] Evans DJ Holian BL. The nose–hoover thermostat. J Chem Phys (1985) 83(8):4069–74. doi: 10.1063/1.449071

[38] Martyna GJ Tobias DJ Klein ML. Constant pressure molecular dynamics algorithms. J Chem Phys (1994) 101(5):4177–89. doi: 10.1063/1.467468

[39] Mahajan S Kode V Bhojak K. Immunodominant T-cell epitopes from the SARS-CoV-2 spike antigen reveal robust pre-existing T-cell immunity in unexposed individuals. Sci Rep (2022) 11:13164. doi: 10.1038/s41598-021-92521-4 PMC8222233 34162945

[40] Hayashi K Morooka N Yamamoto Y Choi S Ohtsubo E Baba T. Highly accurate genome sequences of the Escherichia coli K-12 strains MG1655 and W3110. Mol Syst Biol (2006). doi: 10.1038/msb4100049 PMC1681481 16738553

[41] Ahmad SU Khan MS Jan Z Khan N Ali A Rehman N. Genome wide association study and phylogenetic analysis of novel SARS-COV-2 virus among different countries. Pak J Pharm Sci (2021) 34 (4). 34799302

